# Multifamily therapy in difficult-to-treat depression: an integrated and promising approach to rethinking clinical strategies

**DOI:** 10.3389/fpsyt.2024.1484440

**Published:** 2024-10-31

**Authors:** Walter Paganin

**Affiliations:** School of Dottorate in Neuroscience University of Rome Tor Vergata, Tor Vergata University, Rome, Italy

**Keywords:** difficult to treatment depression, multifamily therapy, difficult to treatment depression management, multifamily therapy-based mental health interventions, multidisciplinary approaches in depression management, innovative therapeutic approaches

## Introduction

### The complexity of DTD and the multidisciplinary approach

In recent years, the concept of Difficult-to-Treat Depression (DTD) has gained attention as a complex clinical problem that requires a multidimensional therapeutic approach. Unlike Treatment-Resistant Depression (TRD), which primarily focuses on the lack of response to pharmacological treatments, DTD acknowledges the importance of psychological, familial, social, and environmental dynamics in determining treatment efficacy (1, 2). Traditionally, academic focus was directed towards the challenge posed by TRD, characterized by the lack of response to standard pharmacological therapies. In contrast, DTD necessitates an integrative approach that considers not only pharmacological treatments but also psychotherapies, neurostimulation techniques, and social and occupational interventions. The definition of DTD is gradually replacing that of TRD both methodologically and semantically (3, 4). While the primary factor in TRD is the poor or absent response to psychopharmacological treatment, DTD centers on elements that negatively interfere with the course of depressive disorder, such as psychiatric and medical comorbidities, the presence of childhood trauma, symptom variability—particularly anhedonia and anxiety—concurrent substance abuse, the use of various failed therapeutic strategies, family history, and adherence issues (5). The formal and structural characteristics of DTD are thus represented by the co-occurrence of clinical and developmental conditions that negatively impact the severity of depressive psychopathological manifestations. This new perspective, which encompasses psychosocial, biological, and interactive aspects, paves the way for an integrative model of therapeutic governance. Recently proposed by a consensus conference of numerous academic experts, this vision presents a revised clinical framework that includes treatment resistance and suggests the adoption of multidisciplinary, personalized, and diversified therapeutic approaches (3). DTD presents a significant challenge, and the integration of psychotherapeutic models involving families can enhance clinical outcomes by providing greater patient support and involving families in the therapeutic process, thereby creating an environment conducive to treatment and relapse prevention. The characterization of DTD suggests a substantial cultural shift to be incorporated into both clinical practice and treatment, highlighting the multifactorial nature of the disorder and the necessity to consider the various contributing factors (1). Managing DTD requires a complex multidisciplinary approach that extends beyond the mere evaluation of pharmacological treatments, including psychotherapies, neurostimulation techniques, and social and occupational interventions aimed at improving treatment adherence and effectiveness. This approach also focuses on enhancing symptom self-management skills and integrating healthcare providers, patients, families, and society (4, 6). In the treatment of DTD, several factors must be considered: those that interfere with the efficacy of pharmacological and psychotherapeutic treatments, those that intensify or prolong depressive symptoms, which must be addressed with specific therapeutic interventions, and psychiatric and medical comorbidities that require specific therapies to be effectively managed. NICE guidelines recommend that patients with DTD, particularly those with moderate to severe depression, should receive psychotherapy as an adjunct or alternative to pharmacological treatment, based on the principle that ineffective treatments should be discontinued (7). Furthermore, a 2019 review already highlighted that the combination of psychotherapy and pharmacotherapy is, on average, more effective than pharmacotherapy alone (8). A better understanding of DTD and the need for patient-specific therapeutic choices can significantly improve clinical outcomes. Moreover, the use of multifamily therapies could represent an innovative and promising approach in the management of DTD.

## Implementation of MFT in the management of DTD

Alongside new pharmacological treatments and neurostimulation, various forms of psychotherapy have shown potential in the treatment of DTD, especially in the presence of comorbidities such as childhood trauma and personality disorders. The Cognitive Behavioral Analysis System of Psychotherapy (CBASP), developed for chronic depression, is particularly focused on interpersonal issues stemming from early trauma and has demonstrated some efficacy, even for patients with DTD and a history of childhood trauma (9). Other promising therapies include Mindfulness-Based Cognitive Therapy (MBCT), a group intervention program originally developed to prevent depression relapse, which may also be relevant for patients with DTD, and Schema Therapy, which has proven particularly useful for patients with pronounced personality traits and emotional trauma from childhood. Although still relatively new in the context of DTD, Schema Therapy has shown promising results in preliminary studies, with significant positive effects on patients with chronic and difficult-to-treat depression (10). From the perspective of DTD, the primary objective of treatment is the optimal control of symptoms, enhancement of quality of life, and the initiation of a personalized program that takes into account the patient’s history, comorbidities, and individual needs. Unfortunately, one of the intrinsic limitations of DTD is the lack of clinical studies specifically focused on this condition. This limitation is likely due to the complexity and still evolving nature of DTD as a clinical entity. However, by analogy, numerous studies have demonstrated the efficacy of MFT in treating depression complicated by comorbidities (11–13). These studies provide significant empirical evidence supporting the use of MFT in complex depressive conditions, which share similar clinical features with DTD (14, 15). The integration of Multifamily Therapy (MFT) into the management of DTD can represent a significant advancement, as these therapies facilitate also active family involvement, creating an environment conducive to healing and relapse prevention. This addition marks a fundamental shift in the field of depression, moving the focus from solely pharmacological response to a comprehensive evaluation of the patient and their life system. The implementation of multifamily therapy in the treatment of difficult-to-treat depression can be a substantial step forward in the care of these patients. MFT, through its ability to actively engage families, creates a therapeutic environment that promotes change and healing (16). These therapies facilitate the expression of emotions and improve coping skills for both patients and their families. This is particularly relevant in DTD, where the presence of psychiatric comorbidities and failed treatments necessitates structured and continuous support.

### Multifamily therapy: an integrated approach

**Figure 1:**
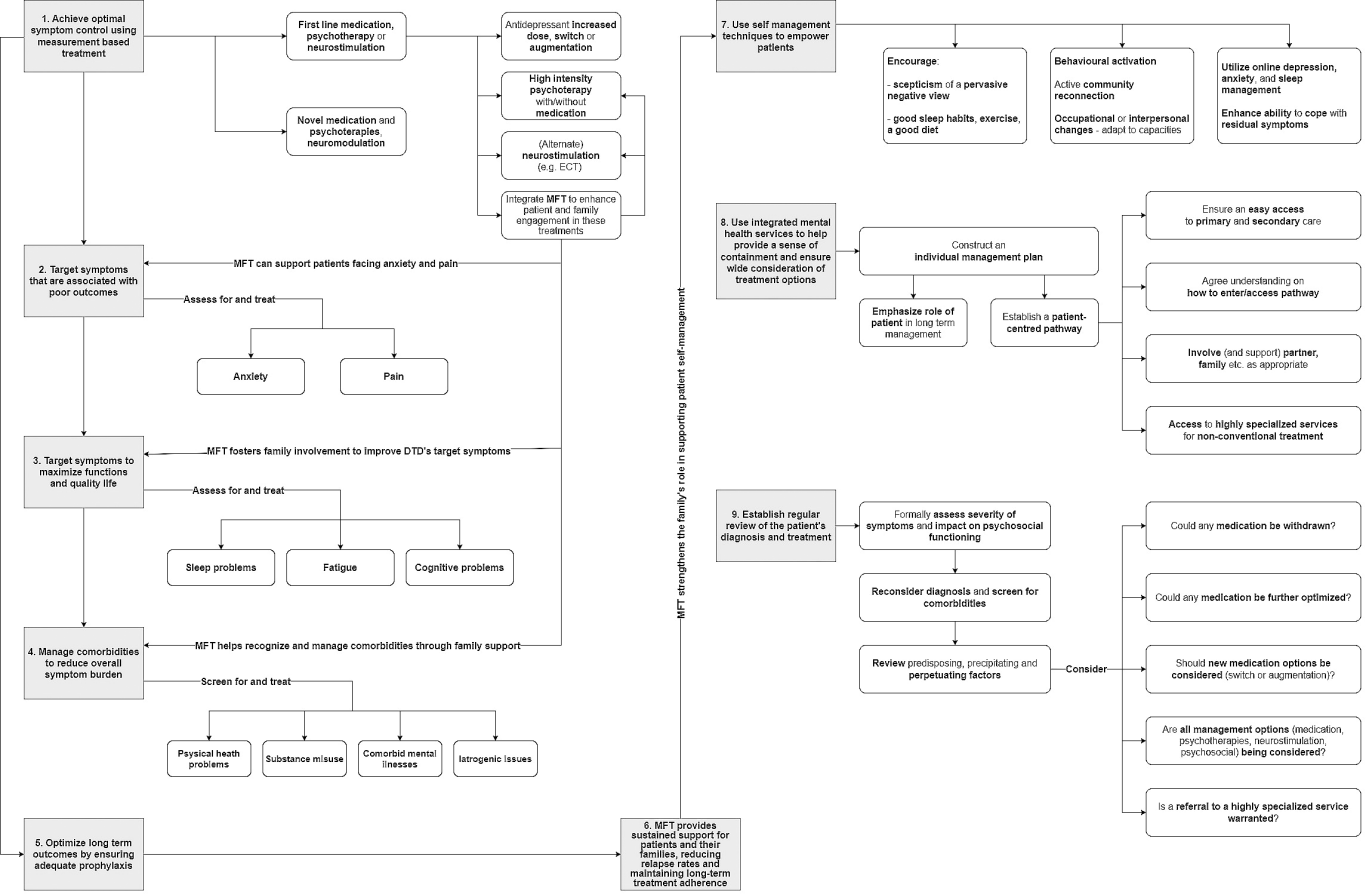
How MTF can be integrated into the management of DTD (from 3), adapted.

Multifamily Therapy involves patients and family members from multiple families, working together with mental health professionals using a variety of approaches and techniques. These groups provide a specific and transformative therapeutic intervention for psychological distress, facilitating change through individual, familial, and inter-familial relational interactions during group sessions (17). Initiated by Peter Laqueur in the 1960s in North America, and almost simultaneously in Argentina by Jorge Garcia Badaracco, who developed multifamily psychoanalysis groups with a psychoanalytic perspective, MFT have evolved over time up to the present day. Tracing the development of MFT is complex due to its heterogeneity. MFT employs diverse techniques based on various theoretical models, including systemic, psychoanalytic, Gestalt, cognitive-behavioral, psychodramatic, and psychoeducational approaches (18). These interventions aim to create new perspectives for change within intra- and inter-familial and generational contexts. The heterogeneity of group composition facilitates the development of “multiple transferences,” further enhanced by the participation of professionals with different backgrounds (19). In this context, it becomes easier to discuss topics that are typically more difficult to address in other therapeutic settings (20). MFT can vary in structure, frequency, number and duration of sessions, participants, issues addressed, group objectives, roles of therapists, therapeutic settings, techniques, and approaches used. There is no single classification for multifamily interventions, and MFT settings can vary significantly in duration, with sessions ranging from three hours to marathon sessions spanning one or more days. The frequency of sessions may be daily, weekly, biweekly, or monthly. Some practitioners limit participation to relatives of at least two generations, while others accept patients alone or with significant representatives. The number of therapists and co-therapists varies, although two is generally considered ideal. Some groups allow new families to join during the therapeutic process, while others maintain a closed group structure (21). Additionally, some therapists have extended multifamily groups to include neighbors, coworkers, relatives, and friends (22). MFT has been developed and applied in various contexts and modalities globally over the years, based on the idea that psychotherapy, family support, and psychoeducation are fundamental in addressing mental disorders. However, MFT is distinct from family-focused therapies, which primarily aim to improve immediate family dynamics by directly addressing relational issues between family members of the individual patient. These interventions focus on correcting specific relational problems that negatively impact the patient’s mental health (23). In contrast, MFT involves multiple families and professionals, creating a collective support environment where not only individual family dynamics are addressed, but also shared experiences among families are valued, fostering solidarity between them. Similarly, psychoeducational groups, although effective in providing information to patients and their families, tend to concentrate more on education about the disease and management strategies, rather than the active and dynamic interactions that occur during MFT sessions. MFT is more interactive and relational, promoting behavioral changes and improvements in psychosocial functioning through dialogue and exchanges between participating families (17). This therapeutic modality involves multiple families sharing similar experiences, creating a network of support and mutual understanding. Multifamily groups can facilitate the expression of emotions and improve coping skills, reducing the family burden associated with mental illness (24). MFT offers several specific benefits for the treatment of depression, due to its ability to actively involve patients and families, thereby creating a therapeutic environment conducive to healing (25, 26). Several studies have long demonstrated how MFT has been successfully implemented in the clinical practice of depression (27–29). This approach is particularly valuable for DTD, given the multifactorial nature of the disorder, which includes medical and psychiatric comorbidities, childhood trauma, and social dysfunctions. By addressing these elements collectively, MFT can create a more supportive and effective therapeutic context see **Figure 1**.

The objectives of Multifamily Therapy in the management of Difficult-to-Treat Depression should focus on:

- Enhancing Treatment Adherence: By actively involving families in the therapeutic process, MFT aims to improve patient adherence to treatment plans, thereby increasing the overall effectiveness of the interventions.
- Reducing Relapse Rates: MFT provides continuous support and coping strategies through family involvement, helping to prevent relapses and maintain long-term remission.
- Improving Social and Emotional Functioning: MFT seeks to enhance patients’ social skills and emotional regulation by creating a more empathetic and supportive family environment.
- Addressing Childhood Trauma: Many patients with DTD have histories of childhood trauma that can complicate treatment. MFT offers a safe space to address these issues and integrates trauma-informed care into the therapeutic process.
- Managing Psychiatric Comorbidities: MFT helps in identifying and managing co-occurring psychiatric disorders, ensuring a comprehensive approach to mental health care.
- Building Resilience and Coping Skills: Through shared experiences and mutual support, families can develop greater resilience and improved coping mechanisms to better manage the challenges of DTD.

Unfortunately, current limitations include the lack of large-scale randomized clinical trials, which makes it difficult to generalize the results obtained in smaller clinical settings. Furthermore, it would be advisable to design longitudinal studies to evaluate the long-term effects of MFT, including in patients with DTD. Suggested methodologies for future studies should incorporate neuroimaging techniques to observe brain changes associated with MFT, along with more sophisticated measures to assess psychological and social outcomes of treatment. I believe that these lines of research can provide new insights into the mechanisms of MFT and enhance its clinical applicability. Ongoing research in this field will be crucial to consolidating the effectiveness of these interventions and developing practical guidelines for mental health professionals.

## Discussion

DTD presents a complex therapeutic challenge that necessitates an integrated and multidimensional approach. In recent years, the growing focus on DTD has led to a redefinition of the concept of treatment-resistant depression, shifting the emphasis from a sole focus on lack of response to pharmacotherapy to a broader perspective that also considers the psychological, social, and environmental dynamics of patients. In this context, MFT may emerge as a promising intervention capable of addressing the multiple facets of DTD through the active involvement of families in the therapeutic process. The integration of MFT in the treatment of DTD may constitute a significant innovation compared to other therapeutic approaches. It not only aims to reduce depressive symptoms but also seeks to improve the patient’s overall functioning, including social and emotional aspects, which is another key goal in the care of DTD. By involving families in the care process, MFT promotes greater adherence to treatment, reduces relapse rates, and provides continuous support—critical elements for patients with DTD. The complexity of DTD often includes childhood trauma, psychiatric comorbidities, and a history of treatment failures, requires targeted interventions that go beyond conventional pharmacological therapies. In this sense, MFT offers a unique opportunity to address these issues in an integrated manner. MFT does not exclude the concomitant practice of other individual psychotherapies, such as the Cognitive Behavioral Analysis System of Psychotherapy, Mindfulness-Based Cognitive Therapy, and Schema Therapy, which have been shown to be effective in the management of DTD. Rather, it offers benefits not only by strengthening the individual psychotherapeutic intervention but also by positively impacting the entire course of treatment (see **Figure 1**). However, it is important to note that the practice of MFT in the treatment of DTD is not yet fully established, and future research should focus on deepening the understanding of these interventions by developing practical guidelines to guide mental health professionals in the application of MFT and in the personalization of treatments based on the specific needs of patients. The importance of adequate training for therapists involved in MFT should also be emphasized to ensure the maximum efficacy of these interventions. The integrative approach proposed by these interventions addresses the need for a cultural shift in the management of depression, recognizing the importance of the familial and social context in treating DTD. This paradigm reflects a deeper understanding of the multifactorial nature of DTD and paves the way for therapeutic interventions that extend beyond the mere administration of medications, promoting a holistic vision of patient care. The management of DTD requires frequent, in-depth clinical reassessments to identify the factors contributing to treatment resistance and to address them with specific integrated interventions, including pharmacological, psychotherapeutic, and somatic therapies. The implementation of MFT in the treatment of DTD represents a significant advancement in the care of this condition within the current clinical and scientific landscape.

In conclusion, a clear definition of DTD and its operational criteria is essential to guide future research and clinical approaches, promoting higher quality care based on the patient’s history, comorbidities, and contingent needs. It remains crucial to continue investigating, the factors contributing to DTD and the adoption of integrated and personalized interventions, thereby improving clinical outcomes for patients and reducing the overall burden of the disease for their families.
